# PMMA bone cement with L-arginine/nano fish bone nanocomplex for apatite formation

**DOI:** 10.1098/rsos.231694

**Published:** 2024-03-27

**Authors:** Gessica Aurel Khoman, Muhammad Harza Arbaha Kalijaga, Nuning Aisah, Riastuti Fidyaningsih, Jarot Raharjo, Oka P. Arjasa, Ekavianty Prajatelistia

**Affiliations:** ^1^ Materials Science and Engineering Study Program, Faculty of Mechanical and Aerospace Engineering, Institut Teknologi Bandung, Jl. Ganesha 10, Bandung 40132, Indonesia; ^2^ Advanced Material Research Center, National Research and Innovation Agency (BRIN), Kawasan Puspiptek Setu Serpong, South Tangerang, Banten 15314, Indonesia

**Keywords:** nano fish bone, PMMA, bone cement, hydroxyapatite, L-arginine, simulated body fluid

## Abstract

Bone cement is one of the materials used in orthopaedics that serves various functions, such as binding bone implants, replacing damaged bones and filling spaces within bones. Various materials have been used to synthesize bone cement, and one promising material for further research is fish bone waste-based bone cement. This study investigates the potential of fish bone waste-based bone cement by incorporating nano fish bone (NFB) and L-arginine (L-Arg) protein into polymethyl methacrylate (PMMA) to examine apatite growth. NFB derived from the *Salmo salar* fish positively influences osteoblast cell proliferation and differentiation, while L-Arg enhances biocompatibility and antibiotic properties. The NFB/L-Arg combination holds promise in accelerating new bone formation and cell growth, both of which are crucial for fracture healing and bone remodelling. Tensile strength tests reveal the superior performance of BC-PMMA-1-NFB/L-Arg (36.11 MPa) compared with commercial PMMA (32 MPa). Immersion tests with simulated body fluid (SBF) solution for 7 days reveal accelerated apatite layer formation, emphasizing the potential benefits of NFB/L-Arg in bone cement applications.

## 1. Introduction

As the world’s population is growing, an increasing number of bone diseases and injuries are becoming public health issues. According to the data published by the Global Burden of Diseases, there were approximately 178 million new cases of bone fractures worldwide in 2019 [1]. These cases are primarily caused by trauma, inflammation and osteoporosis [2]. One solution to address this problem is the use of bone implants. Ongoing studies on bone implants aim to extend the lifespan of these implants and minimize the need for additional surgeries. One of the components of bone implants is bone cement.

Bone cement is a material employed to stabilize implants and ensure uniform mechanical distribution between the implant and the bone [3]. Apart from its use in hip implants, bone cement is also used in joint reconstruction, cranioplasty, aneurysm investment, fixation of pathological fractures, blind eyeballs and bone replacement [4]. Bone cement comprises two phases: a powder phase and a liquid phase. When these components are mixed and stirred, polymerization occurs, resulting in the formation of a plastic paste that sets once implanted in the body. Consequently, the microstructure and mechanical properties of bone cement are influenced by the chemical composition and concentration of each constituent [5].

Polymethyl methacrylate (PMMA) is the most commonly used material in bone cement due to its favourable mechanical properties and simple operation procedure [6]. It is a lightweight synthetic polymer and a cost-effective alternative when high-strength materials are not required. It is extensively used in various biomaterial applications, including bone cement, lenses, bone substitutes and drug delivery systems [7]. Emulsion polymerization is one of the most popular PMMA synthesis methods [8]. This method involves an emulsification process with the aid of surfactants. Surfactants reduce interfacial tension and can form micelles to regulate particle size [9]. The emulsion method uses the principle of emulsification, where hydrophilic monomers are dispersed into an aqueous surfactant solution. A shear force is applied to the monomer mixture with the surfactant for a specific duration to achieve a uniformly dispersed monomer. The emulsion of monomer and surfactant solutions results in a stable solution due to the absorption of surfactants at the water–monomer interface. Sodium dodecyl sulfate (SDS) is the most used anionic surfactant because it provides greater rigidity than other surfactants when preparing polymer granules. Anionic surfactants can be used to reduce the hydrophobicity of polymer granules and stabilize the suspension charge. PMMA is a polymer with a hydrophobic surface and its hydrophilic properties need to be enhanced to increase hydrophilicity and ensure good adhesion to other substrates. Therefore, anionic surfactants are necessary for decreasing hydrophobicity and enhancing hydrophilicity [10–12].

However, PMMA still has notable limitations, such as its inability to bond with bone tissue. When implanted *in vivo*, PMMA exhibits poor integration with the surrounding bone tissue and does not facilitate bone cell adhesion and growth [6,13]. Based on these problems, the addition of other materials is essential. One common substance used for bone repair is hydroxyapatite (HA). Since hydroxyapatite (Ca_10_(PO_4_)_6_(OH)_2_) has chemical characteristics that are comparable to those of the mineral found in mammalian bones and hard tissues, it is a well-known biomaterial that is frequently used in medical applications such as dental paste, bone cement and implants [14,15]. HA can be manufactured synthetically using calcium and phosphate sources or extracted from natural resources. An alternative for producing HA is to obtain it from animal by-product sources such as mammal bones, fish bones, fish scales, egg shells and shellfish. The formation of natural HA sourced from animal by-products involves an extraction process that aims to remove organic materials from the mineral matrix to obtain HA directly. Some of these extraction processes include calcination methods, alkaline hydrolysis, precipitation, hydrothermal or a combination of these techniques [16]. HA extracted from animal by-products has promising potential for various biomedical applications because it is biocompatible and bioactive [17]. Fish bones are an alternative that can be used to overcome the shortcomings of PMMA.

On the other hand, approximately greater than 91 million tons of fish and shellfish are consumed every year in the world [18]. Some by-products, about 40–50% of the total fish, are removed as waste during processing. Fish bones are a significant by-product of fish processing and are usually considered impractical and worthless waste [19]. Therefore, using fish bone waste will solve two problems at once: improving the performance of PMMA bone cement and overcoming the waste problem to support sustainable environmental development. It is a potential strategy to use the waste produced by the fishing industry to isolate HA using straightforward, low-cost and biologically safe techniques that do not involve the use of chemical processing chemicals [20]. Trace levels of ions including Na^+^, Mg^2+^, Zn^2+^ and K^+^ are typically present in HA which is recovered from fish bones [18]. The natural HA has various benefits due to the presence of these trace ions. It has been noted that these trace ions aid in the processes of bone regrowth and creation [15].

Fish bones as a source of HA are supplemented with L-arginine protein (L-Arg) to promote the development of apatite in PMMA bone cement. L-Arg is a semi-essential amino acid required for various biological processes, including osteogenic and angiogenic activities [21,22]. It is a crucial building block for the production of proteins and other compounds such as nitric oxide (NO), ornithine, proline and polyamines [23]. NO has been shown to promote bone formation and limit bone resorption. Therefore, L-Arg contributes to angiogenesis, which is necessary for bone remodelling and fracture healing [22].

This research focuses on producing PMMA bone cement using nano fish bone (NFB) derived from fish bone waste, specifically from the *Salmo salar* fish. NFB is rich in calcium, phosphorus, protein, fat and other nutrients, which contribute to its ability to enhance bone regeneration through the principles of osteoimmunology [24]. The novelty of this research lies in the combination composition of NFB from the *Salmo salar* fish with the L-Arg protein for the formation of apatite in PMMA bone cement. The apatite formation promoted by the addition of NFB/L-Arg is expected to accelerate new bone formation, a process necessary for fracture healing and bone remodelling. The addition of arginine to the polymer is also known to improve biocompatibility and provide an antimicrobial effect, offering resistance against the attachment of gram-positive and gram-negative bacteria [25]. The effects of NFB and NFB/L-Arg addition on the mechanical strength, mass change and mass increase rate will be evaluated in this study.

## 2. Methodology

### 2.1. Materials

Methyl methacrylate (MMA, 99% purity), SDS (greater than 99% purity), potassium peroxodisulfate (KPS, less than or equal to 100% purity), benzoyl peroxide (BPO, greater than or equal to 70% to less than 99% purity) and *N*-*N*-dimethyl-*p*-toluidine (DMPT, less than or equal to 100% purity) by Merck (Darmstadt, Germany).

### 2.2. PMMA synthesis

The emulsion method was used for PMMA synthesis, which involves an emulsification process with the aid of surfactants. The emulsion polymerization process starts by dissolving the monomer into a surfactant solution. MMA was used as the monomer, potassium persulfate (KPS) was used as the initiator and SDS was used as the surfactant.

The weight percentage ratio of MMA to water was 20 wt%. The ratio of KPS to MMA was 1 wt% [26]. The variation observed was in the ratio of SDS to MMA, specifically 0, 1, 3 and 5 wt%. Table 1 presents the names of the samples and their mass specifications for PMMA synthesis.

**Table 1.** Mass specifications of materials used in PMMA synthesis.

sample	MMA (g)	SDS (g)	Aqua DM (g)	KPS (g)
PMMA-0-SDS	40	0	200	0.4
PMMA-1-SDS	40	0.4	200	0.4
PMMA-3-SDS	30	0.9	150	0.3
PMMA-5-SDS	30	1.5	150	0.3

### 2.3. Nano fish bone preparation

NFB preparation was carried out using the *Salmo salar* species. The preparation of NFB samples was performed by following the methods described by Yin *et al*. [27]. The fish was cut into 5 cm lengths and subjected to pressing for 1 hour at 121°C. Subsequently, the samples were rinsed with water to remove residual fat and meat. Next, the fish bones were crushed using either a meat grinder or a blender through the milling method at 1000 r.p.m. for 5 minutes to reduce the particle size. Before milling, they were mixed with cold water in a ratio of 1:0.3 to cool down the sample and minimize the heat generated during grinding. The resulting sample, in the paste form, was dried in an oven at 105℃ for 6 hours. After drying, the sample was ground using a mortar and passed through a 200-mesh sieve. After that, the samples were characterized by functional group analysis, particle size analysis and Ca:P content analysis in fish bones.

### 2.4. Bone cement synthesis

The formation of bone cement referred to the journal articles on manufacturing PMMA bone cement with HA additives from Kang *et al*. [28]. PMMA powder, MMA monomer mixed with 1 wt% BPO and 1 wt% DMPT were weighed in a 2:1 ratio of PMMA and MMA. Then, the dry ingredients (PMMA, BPO and L-Arg/NFB) were mixed with the liquid ingredients (DMPT and MMA) and the mixture was placed in the mould and left for the curing process to be completed. After curing, the samples were characterized to study the properties of the resulting bone cement. The concentration of the L-Arg/NFB nanocomplex used was 2.5 wt% for each of the masses of PMMA used. The mass composition of each material can be seen in table 2.

**Table 2.** Mass specification for bone cement.

sample	PMMA (g)	MMA (g)	BPO (g)	DMPT (g)	NFB (g)	L-arginine (g)
BC-PMMA-0	3 (PMMA-0-SDS)	6	0.12	0.06	—	—
BC-PMMA-1	3 (PMMA-1-SDS)	6	0.12	0.06	—	—
BC-PMMA-3	3 (PMMA-3-SDS)	6	0.12	0.06	—	—
BC-PMMA-5	3 (PMMA-5-SDS)	6	0.12	0.06	—	—
BC-PMMA-0- NFB	3 (PMMA-0-SDS)	6	0.12	0.06	0.075	—
BC-PMMA-0-NFB/L-Arg	3 (PMMA-0-SDS)	6	0.12	0.06	0.075	0.075
BC-PMMA-1- NFB	3 (PMMA-1-SDS)	6	0.12	0.06	0.075	—
BC-PMMA-1-NFB/L-Arg	3 (PMMA-1-SDS)	6	0.12	0.06	0.075	0.075

### 2.5. Characterization

Solid content analysis, Fourier-transform infrared (FTIR), particle size analysis (PSA) and scanning electron microscopy (SEM) characterization studies were performed to determine the effectiveness of the polymerization, structure, particle size and morphology of PMMA. The solid content was calculated to determine the percentage of the polymer in PMMA samples resulting from the polymerization process. Calculations were performed using equation (2.1) [Disp-formula uFD1][29].


(2.1) $$Solid\%=\left[W_{f}\right]/\left[W_{s}\right],$$

where *W*_f_ is the weight of the dried emulsion and *W*_s_ is the weight of the undried emulsion. FTIR characterization was carried out using Bruker Alpha II, PSA characterization with a nanoparticle analyser Horiba SZ-100 (Japan) and scanning electron microscopy–energy dispersive X-ray spectroscopy (SEM-EDS)characterization with Hitachi SU3500 (Japan).

NFB analysis was performed using FTIR, PSA and EDS. X-ray diffraction (XRD) characterization, tensile testing and simulated body fluid (SBF) immersion testing were carried out for bone cement characterization. The tensile testing was carried out using GOTECH AI-7000-S (China) following the ASTM D638 standard. Immersion testing was carried out using a method reported by Kokubo and Takadama [30].

### 2.6. Statistical analysis

All experiments were performed in triplicate unless otherwise stated. The statistical comparison was assessed using one-way ANOVA with a Tukey–Kramer comparison test (figure 8) using Origin version 10.1 (OriginLab Corporation). The results were considered significant when p≤0.05 (*), very significant when p≤0.01 (**) and highly significant when p≤0.001 (***).

## 3. Results

### 3.1. PMMA solid content analysis and structure

After emulsion polymerization and the production of PMMA with four different SDS additions, solid content analysis was performed on all PMMA samples. The results of the solid content analysis, presented in table 3, were used to assess the suitability of the PMMA content formed with varying percentages of MMA used during polymerization. MMA used in PMMA synthesis was 20 wt% relative to the solvent. The PMMA-0-SDS sample achieved complete polymerization, while the other three samples exhibited polymerization levels exceeding 85%. This indicates that the polymerization of all PMMA samples effectively converted MMA into PMMA.

**Table 3.** Solid content results of PMMA-0-SDS, PMMA-1-SDS, PMMA-3-SDS and PMMA-5-SDS.

sample	solid content (%)
PMMA-0-SDS	20.14
PMMA-1-SDS	16.35
PMMA-3-SDS	18.29
PMMA-5-SDS	16.97

**Figure 1.**
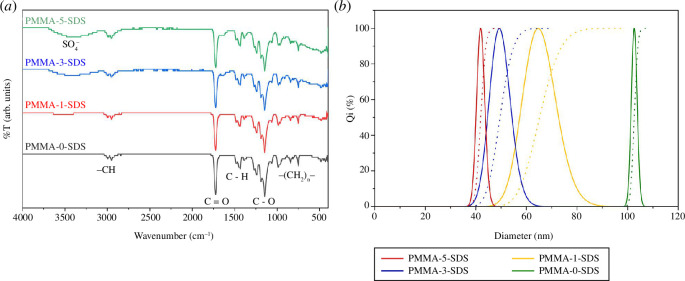
(*a*) FTIR results and (*b*) PSA results of PMMA-0-SDS, PMMA-1-SDS, PMMA-3-SDS and PMMA-5-SDS.

FTIR analysis was performed on PMMA-0-SDS, PMMA-1-SDS, PMMA-3-SDS and PMMA-5-SDS. The results of the FTIR analysis of PMMA were compared with the structure of PMMA, as shown in figure 1*a*. The FTIR results correspond to the groups in the PMMA structure. In the FTIR spectra of PMMA-1-SDS, PMMA-3-SDS and PMMA-5-SDS, there are peaks at a wavelength of approximately 3400 cm^−1^. These peaks indicate the presence of SO_4_^–^ in PMMA due to the addition of SDS.

### 3.2. PMMA particle size and morphology

According to figure 1*b*, the addition of SDS during PMMA synthesis leads to a decrease in the particle size of the produced PMMA as the percentage of SDS increases. The PMMA-5-SDS sample exhibited an average particle diameter of 42.1 nm, whereas the PMMA-0-SDS sample had a particle diameter of 102.8 nm. Surfactants play a crucial role in controlling the particle size. In the presence of surfactants, the particle size of PMMA can be adjusted to achieve a uniform size distribution. Surfactants can form micelles during emulsion polymerization, resulting in particles with diameters of approximately 10 nm, depending on the treatment during the polymerization process [31]. SDS, as a surfactant, can reduce the surface tension between the air phase and the oil phase in the emulsion system, so that PMMA particles are more easily dispersed and stable. Lower surface tension facilitates the formation of small particles of more uniform size. Therefore, the particle size of PMMA decreases with an increasing SDS concentration up to a specific concentration value [32]. The data indicate that an increased SDS content leads to smaller and more uniformly sized PMMA particles.

**Figure 2.**
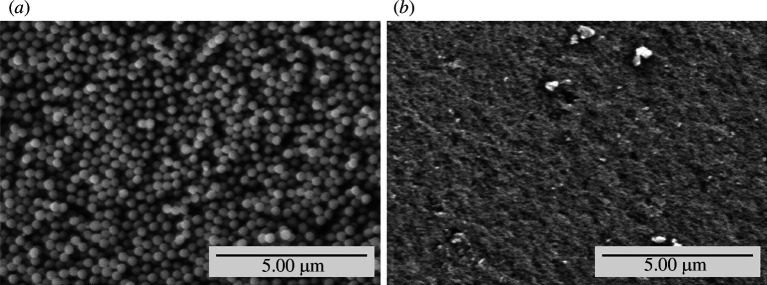
SEM characterization of (*a*) PMMA-0-SDS and (*b*) PMMA-5-SDS.

SEM morphological characterization was performed on PMMA-0-SDS and PMMA-5-SDS. These two samples were selected to study the morphological differences in PMMA samples with and without SDS. As shown in figure 2, PMMA-0-SDS produced uniform round grains, while PMMA-5-SDS shows that the PMMA particles are agglomerated, so the grains are not clearly visible. This is because PMMA-5-SDS has a very small particle size, so agglomeration occurs easily due to the high surface area of nanoparticles and attractive solid interactions between the particles [33]. The agglomerated nanoparticles usually have primary bonds in the form of van der Waals, electrostatic and magnetic forces, and capillarity effects in the case of wet particles [34]. In this case, the PMMAs have van der Waals bonding and result in agglomeration. Agglomeration can cause a decrease in mechanical properties and cytotoxicity effects due to nanoparticle sedimentation in cells [35].

### 3.3. Nano fish bone structure

**Figure 3.**
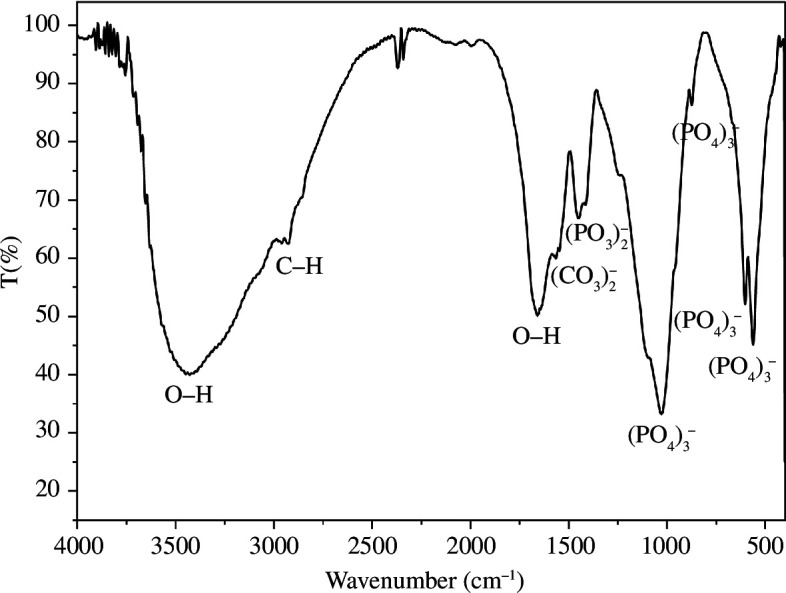
FTIR spectrum of the NFB sample.

The identification of functional groups was performed based on references that correspond to the peaks of the FTIR graph in the NFB sample as shown in figure 3. After comparing the obtained FTIR results with the reference FTIR results of HA from Arunseshan *et al*. [36], it was observed that all the functional groups characteristic of HA were present. In reference, the peak around 3568 cm^−1^ confirmed the presence of a hydroxyl group. Additionally, the presence of carbonyl and phosphate groups was also confirmed. Based on these findings, it can be concluded that the structure of NFB is similar to that of HA.

### 3.4. Nano fish bone particle size, morphology and Ca:P ratio

**Figure 4.**
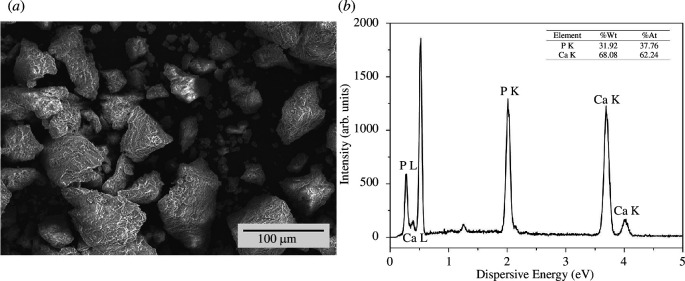
(*a*) SEM image and (*b*) EDS results of the NFB sample.

The particle size in the NFB sample was measured using PSA, and the average size value was 757.5 nm with 0.527 polydispersity index (PDI). In the SEM results of NFB, as shown in figure 4*a*, the agglomeration of the NFB particles is observed. This agglomeration leads to non-uniformity in the size of the formed NFB particles. In addition to the agglomeration effect, the PSA results also indicate a relatively large PDI value. A higher PDI indicates a greater degree of particle size non-uniformity.

The EDS characterization focused on examining the Ca:P content of the NFB sample. Based on the results presented in figure 4*b*, the Ca:P content of the NFB sample was found to be 1.65, while that in human bones typically ranges around 1.67. It can be observed that the Ca:P content obtained from NFB preparation closely resembles the Ca:P content of HA found in human bones.

### 3.5. X-ray diffraction analysis of nano fish bone and bone cement

**Figure 5.**
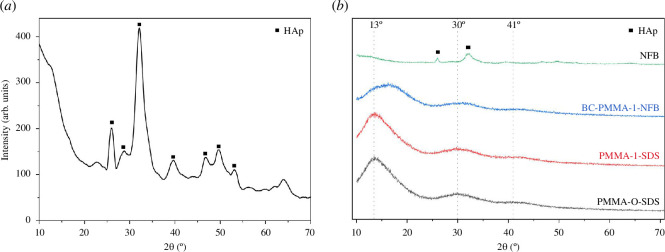
XRD results of (***a***) NFB and (***b***) PMMA-0-SDS, PMMA-1-SDS, BC-PMMA-1-NFB and NFB.

XRD characterization was performed on NFB samples to analyse the phases present, as shown in figure 5*a*. The observed peaks corresponded to the ICDD PDF standard 01-074-0565 and were consistent with the reference [36]. Additionally, the phase analysis using the PROFEX application confirmed the presence of 100% HA. The presence of medium-intensity bands around 26°, high-intensity bands around 32° and low-intensity bands around 28°, 39°, 46°, 49° and 53° confirmed the presence of HA [21].

**Figure 6.**
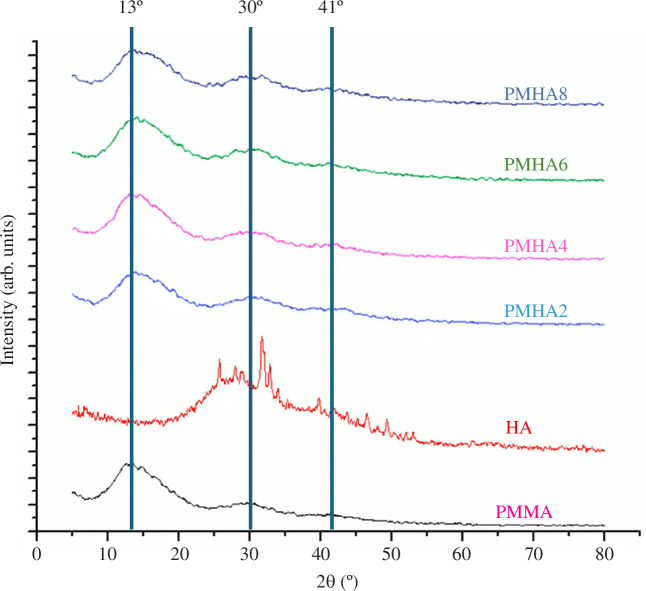
XRD results of PMMA, PMMA/HA and HA from the reference [37]. Copyright 2021 MDPI.

In addition to NFB, XRD characterization was also conducted on PMMA and PMMA/NFB bone cement samples to identify the phases formed, which can be seen in figure 5*b*. The XRD peak observed in the PMMA sample corresponds to the peak observed in the reference XRD results of PMMA (figure 6), thereby confirming the presence of PMMA in the sample. The broad peaks observed in the high-intensity region around 13°, as well as the two low-intensity bands around 30° and 41°, indicate the amorphous nature of PMMA. According to the reference, when compared with the XRD results of NFB, the XRD results of PMMA-1-NFB do not show the presence of peaks for NFB. This indicates that the structure of PMMA is not affected by the addition of HA (NFB), and no chemical reaction occurs between PMMA and HA (NFB) [37].

### 3.6. Tensile testing of bone cement

**Figure 7.**
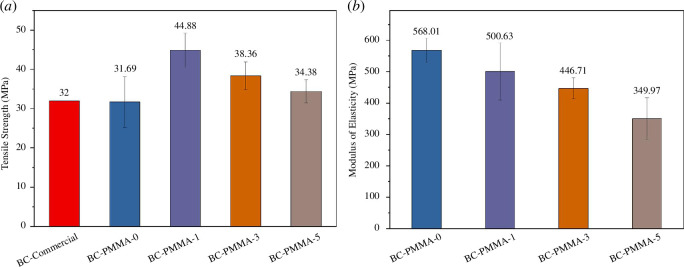
(*a*) Tensile strength and (*b*) modulus of elasticity of PMMA with SDS variation.

Figure 7*a* shows that PMMA-1-SDS has the greatest tensile strength value. From the PSA test results (figure 1*b*), PMMA-1-SDS has the second-largest particle size after PMMA-0-SDS. The smaller the particle size, the higher the mechanical strength of the composite will be [38]. However, from the tensile test results, this did not happen. Judging from the results of SEM morphology (figure 2) of PMMA-0-SDS (the largest particle size) and PMMA-5-SDS (the smallest particle size), in PMMA-0-SDS, the particle shapes of the resulting product are uniform and round. Whereas, in PMMA-5-SDS, the SEM results show agglomeration in the particles, so the appropriate morphology could not be seen. The agglomeration of nanoparticles reduces the potential for improving the mechanical properties of nanocomposites due to the limited interfacial area [30,39]. In addition, agglomeration can produce many defects and stress concentrations in the nanocomposite and damage the sample properties [40]. Thus, from the tensile test results, PMMA-1-SDS produces the highest tensile strength because PMMA-1-SDS with SDS 1 wt% produces an optimum particle size that does not produce agglomeration.

Figure 7*b* shows that as the percentage of SDS concentration in PMMA increases, the modulus of elasticity of PMMA bone cement decreases. However, when compared with cortical bone, which has a modulus of elasticity of 17 GPa, the modulus of elasticity of the PMMA bone cement sample is still far below that of human bone.

**Figure 8.**
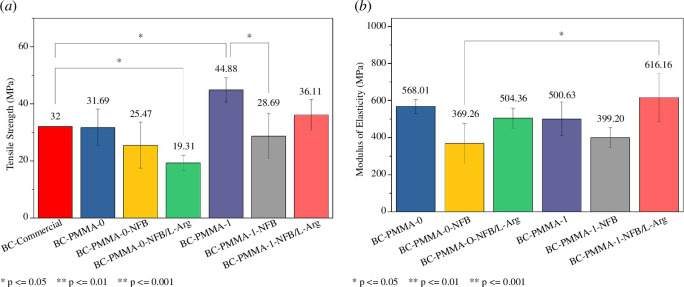
(*a*) Tensile strength and (*b*) elasticity modulus value of BC-PMMA-0 and BC-PMMA-1 with NFB and NFB/L-arginine.

Tensile tests were also carried out on PMMA bone cement with the addition of NFB and NFB/L-Arg using PMMA-0-SDS without surfactant, and PMMA-1-SDS with optimal surfactant concentration. Figure 8*a* shows that the addition of NFB and NFB/L-Arg decreased the mechanical properties of BC-PMMA-0 and BC-PMMA-1. The different coefficients of thermal expansion between PMMA and NFB caused a decrease in mechanical properties due to the addition of NFB. NFB has the same structure as HA, as shown in the FTIR results in figure 3. From the reference, PMMA has a coefficient of thermal expansion of 8.1 × 10^−5^ ℃^−1^, and HA has a coefficient of thermal expansion almost 10 times different of 8.3 × 10^−6^ ℃^−1^. As a result of the difference in the coefficient of thermal expansion, there will be a difference in the volume at the time of making bone cement. In the cooling phase, due to the exothermic polymerization reaction in the formation of bone cement, both PMMA and HA materials will shrink.

Because the shrinkage of the PMMA matrix is more significant than the shrinkage of the HA particles, HA will be trapped and tightly squeezed in the PMMA matrix. As a result, a hoop tensile stress is formed in the bone cement matrix adjacent to the HA particles. This stress simulates the formation of a weak bond between the HA particles and the resulting PMMA matrix. When a load is applied, the HA particles in the continuous PMMA phase constitute the second phase, and the interface between the two phases acts as a grain boundary that resists crack propagation. Friction forces opposite to the initial crack force will appear at the crack edges. The friction force is higher than the hoop tension. When a tensile load is applied, the crack direction is perpendicular to the loading direction. The hoop stress helps separate the crack edges so that the HA-containing bone cement will weaken the tension problems [41].

Although there was a significant decrease in BC-PMMA-1 when NFB was added, its strength increased with the addition of L-Arg. The increase that occurred was able to exceed the tensile strength of BC-Commercial. Furthermore, figure 8*b* shows that the addition of NFB can decrease the modulus of elasticity of bone cement, but when added with L-Arg, the modulus of elasticity increases. This is because L-Arg can interact ionically with NFB [42]. From the tensile strength data obtained, only two samples (BC-PMMA-1 and BC-PMMA-1-NFB-L-Arg) could exceed the tensile strength value of commercial PMMA (CEMEX RX; 32 MPa) [43]. L-Arg can form ionic bonds with HA (NFB) and can improve the mechanical properties of PMMA, supported by the data obtained that BC-PMMA-1-NFB experienced an increase in tensile strength after the addition of L-Arg.

### 3.7. Immersion testing of bone cement

**Figure 9. F9:**
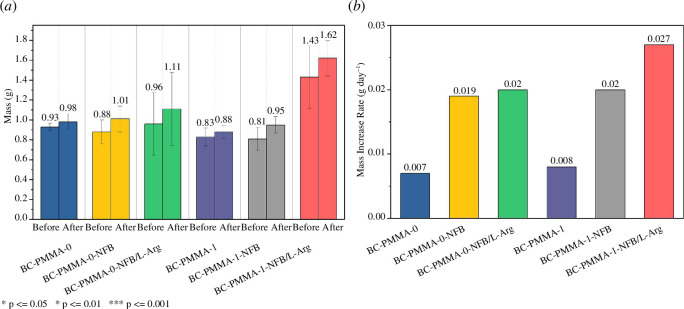
(*a*) Mass before and after the immersion test and (*b*) mass increase rate of the bone cement sample by the SBF immersion test.

Immersion testing was performed on BC-PMMA-0, BC-PMMA-0-NFB, BC-PMMA-0-NFB/L-Arg, BC-PMMA-1, BC-PMMA-1-NFB and BC- PMMA-1-NFB/L-Arg for 7 days to determine the mass changes after immersion in the SBF solution. The results of the immersion test are shown in figure 9*a*. In general, all bone cement samples experienced an average increase in mass after being immersed in the SBF solution. This shows that the initial hypothesis is that NFB (HA) can form an apatite layer from a damp SBF solution. Figure 9*b* shows the mass increase rate of the sample after the immersion test. From the graph, it is noted that the addition of NFB is able to increase the mass increase rate by more than twofold for both BC-PMMA-0 and BC-PMMA-1 samples. Then, in BC-PMMA-0-NFB, with the addition of L-Arg, there was an increase but it was not significant. However, in BC-PMMA-1-NFB, the increase in the mass increase rate appeared quite high when L-Arg was added. In the BC-PMMA-1-NFB/L-Arg sample, the highest mass increase rate was obtained. This cannot be separated from the role of L-Arg, which has superior properties in angiogenesis and bone repair.

### 3.8. Bone cement morphology after immersion

**Figure 10.**
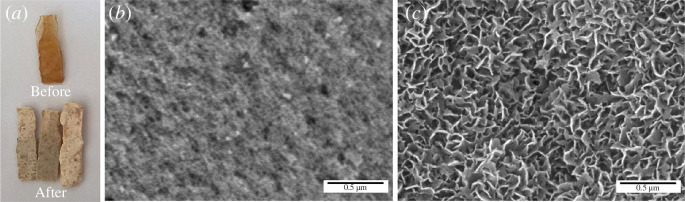
(*a*) Visual appearance before (top) and after (bottom) the immersion test and SEM image of the BC-PMMA-1-NFB/L-Arg sample (*b*) before and (*c*) after the immersion test.

Immersion in SBF solution is a biomineralization process that plays an important role in the bioactivity of bone cement. The process includes nucleation, precipitation and simultaneous growth of an apatite layer on the surface of the SBF sample [40], as explained in the previous chapter. Figure 10*a* [Fig F9] shows visual observations of the samples before and after the immersion test. The colour of the immersed samples becomes whiter and the surface roughness of the bone cement samples becomes coarser than that of non-immersed samples. The colour change to white is due to the increasing number of HA, which has a milky white colour [44]. The coarser appearance also indicates that more HA content can increase cell attachment, cell proliferation and adhesion strength [45]. The interaction of SBF on the substrate was explained by Tanahashi and Matsuda [46] [[]], that the SBF solution has Ca^2+^ and HPO_4_^2–^ ions. The nucleation of apatite formation is initiated by the absorption of Ca^2+^ ions on the surface, which has a negative charge, followed by the uptake of HPO_4_^2–^ ions through ionic interactions with Ca^2+^ and the formation of crystals or CaP nanoparticles. Over time, the accumulation of nanoparticles forms an apatite-like layer on the substrate surface [46]. The efficiency of apatite formation depends on the functional groups present on the substrate surface. Efficiency: H_2_PO_4_ > –COOH > –OH > –NH_2_ > –CH_3_ [47]. It can be seen from the efficiency that the addition of NFB, which contains HPO_4_^2–^, can increase the efficiency of apatite growth, as well as the addition of L-Arg, which has a –COOH functional group. From the results of the immersion test, it can be concluded that the BC-PMMA-1-NFB/L-Arg sample is an optimal bone cement sample for the growth and formation of an apatite layer which is able to promote bone growth.This morphology was obtained because an apatite layer had formed on the sample surface.

Figure 10 shows the morphology of the BC-PMMA-1-NFB/L-Arg sample before and after the immersion test in SBF solution for 7 days. The morphology shows an apatite structure with an aspect ratio c/a of 10.71. After the immersion test, the SEM image in figure 10*c* shows that a coating layer with a needle-like structure covered the sample surface differently from that before immersion as shown in figure 10*b*. This can happen because HA is composed of Ca^2+^, PO_4_^3–^ and OH^–^ molecules on the PMMA surface which functions as a nucleation site and triggers the deposition of an apatite layer through electrostatic interactions between these bioactive molecules [48]. The apatite layer can act as an interface between living hard tissue and the bone cement sample during implantation through efficient bond formation [49]. Then, in the EDS spectrum, shown in table 4, the sample surface has a Ca:P ratio value of 1.65, which is very close to the Ca:P ratio of HA (1.67) in the human body. These results corroborate previous tests that showed increased mass after immersion in SBF solution, indicating that an apatite layer had formed on the sample surface. The presence of apatite indicates that bone cement has superior bone tissue repair capabilities.

**Table 4.** EDS results of the BC-PMMA-1-NFB/L-Arg sample after the immersion test.

element	%Wt	%At
P K	31.88	37.71
Ca K	68.12	62.29

%At stands for atomic percent, while %Wt stands for weight percent.

The claim that bone cement samples with the addition of NFB from salmon bones have superior bone tissue repair capabilities is also strengthened by several related research results. As in the research by Shi *et al*. [50], salmon bones could significantly promote osteoblast viability after 3 and 7 days of incubation. Bone formation ability and cell viability are very important to evaluate the potential of HA as a type of bone replacement material. HA from salmon bones also positively influences osteoblast cell proliferation and differentiation [50]. The same thing was also reported by Venkatesan *et al*. [51], where HA from salmon interacted positively with mesenchymal stem cells (MSCs) and promoted non-toxicity [51]. Based on these findings, HA from salmon bones is an ideal material for bone repair, and bone cement samples that have been synthesized with the addition of L-Arg protein will be an ideal bone cement material for bone replacement or repair.

## 4. Conclusion

PSA characterization reveals that the addition of SDS as a surfactant results in PMMA with smaller particle sizes. Among the samples, PMMA-5-SDS exhibits the smallest particle size, with an average diameter of 42.1 nm. SEM results indicate that PMMA without the surfactant exhibits a morphology of round PMMA grains, while PMMA-5-SDS shows agglomerated PMMA grain morphology due to the small particle size.

The FTIR and XRD results confirm that NFB possesses a chemical structure similar to HA. PSA results indicate that the particle size of NFB is 757.5 nm. EDS results demonstrate that the Ca:P ratio of NFB is similar (1.65) to that of HA in the human body (1.67).

In the BC-PMMA-1-NFB sample, the tensile strength increases after the addition of L-Arg. The BC-PMMA-1-NFB/L-Arg sample exhibits superior tensile strength compared with commercial PMMA. Then, all samples experienced an increase in the mass rate after being immersed in SBF solution for 7 days. The addition of NFB can increase the mass increase rate significantly. The addition of L-Arg was also able to increase the mass increase rate of the BC-PMMA-0-NFB and BC-PMMA-1-NFB samples. BC-PMMA-1-NFB/L-Arg is the most optimal bone cement based on mechanical tests and immersion tests, and has a higher tensile strength than BC-Commercial and has the highest mass increase rate.

## Data Availability

The dataset used in this study is available at Dryad Digital Repository [52].
